# DNA methylation alternation in Stanford- A acute aortic dissection

**DOI:** 10.1186/s12872-022-02882-5

**Published:** 2022-10-29

**Authors:** Yufei Chen, Xu Xu, Zhaoran Chen, Bi Huang, Xiaojian Wang, Xiaohan Fan

**Affiliations:** 1 Department of Cardiology, Fuwai Hospital, National Center for Cardiovascular Diseases, Chinese Academy of Medical Sciences, Peking Union Medical College, Beijing, China; 2 Department of Geriatrics and Gerontology, Beijing Friendship Hospital, Capital Medical University, Beijing, China; 3 Department of Cardiology, The First Affiliated Hospital of Chongqing Medical University, Chongqing, China; 4 Key Laboratory of Pulmonary Vascular Medicine, State Key Laboratory of Cardiovascular Disease, Fuwai Hospital, National Center for Cardiovascular Diseases, Chinese Academy of Medical Sciences and Peking Union Medical College, Beijing, China

**Keywords:** DNA methylation, Epigenetics, Acute aortic dissection, Vascular

## Abstract

**Background:**

Acute aortic dissection (AAD) is a life-threatening cardiovascular disease. Recent studies have shown that DNA methylation may be associated with the pathological mechanism of AAD, but the panorama of DNA methylation needs to be explored.

**Methods:**

DNA methylation patterns were screened using Infinium Human Methylation 450 K BeadChip in the aortic tissues from 4 patients with Stanford-A AAD and 4 controls. Gene enrichment was analyzed by Kyoto Encyclopedia of Genes and Genomes (KEGG) pathway and gene ontology (GO). DNA methylation levels of candidate genes were determined by pyrosequencing in the replication cohort including 16 patients with AAD and 7 controls. Protein expression level of candidate gene was assessed by Western blot.

**Results:**

A total of 589 differentially methylated positions including 315 hypomethylated and 274 hypermethylated positions were found in AAD group. KEGG analysis demonstrated that differentially methylated position-associated genes were enriched in MAPK signaling pathway, TNF signaling pathway and apoptosis pathway, et al. GO analysis demonstrated that differentially methylated position-associated genes were enriched in protein binding, angiogenesis and heart development et al. The differential DNA methylation in five key genes, including Fas, ANGPT2, DUSP6, FARP1 and CARD6, was authenticated in the independent replication cohort. The protein expression level of the Fas was increased by 1.78 times, indicating the possible role of DNA methylation in regulation of gene expression.

**Conclusion:**

DNA methylation was markedly changed in the aortic tissues of Stanford-A AAD and associated with gene dysregulation, involved in AAD progression.

**Supplementary Information:**

The online version contains supplementary material available at10.1186/s12872-022-02882-5.

## Background

Acute aortic dissection (AAD) is a life-threatening disease. Stanford-A AAD, which accounts for almost 75% of AAD cases, is the most frequent type of AAD characterized by sudden onset, rapid progression, and poor prognosis[1–3]. AAD stems from an interplay among many detrimental factors, such as genetic, environmental and epigenetic factors. Genetic is the most common risk factor for the development of AAD, however, it explains only part of the disease mechanisms[4]. The molecular causes of AAD are still not well understood.

Previous studies had identified the differentially expressed genes in AAD patients[5–9]. Epigenetic regulates gene expression without altering the genomic sequences[10, 11]. DNA methylation is a major epigenetic mechanism, which mainly occurs through the linkage of methyl group to the 5′ position of cytosine at cytosine-paired-with-guanine (CpG) dinucleotide sequences[10, 12]. DNA methylation can be mediated by DNA methyltransferases (DNMTs) such as DNMT1, DNMT3A, and DNMT3B[13, 14]. The hypermethylation of DNA could reduce the transcription activity of gene promotor and lead to gene silencing[15, 16]. It was found in our previous study that the protein expression levels of DNMT1 and DNMT3B decreased significantly in AAD samples while the expression of DNMT3a and DNMTL had the decreased trends with no significance, which indicated the potential role of DNA methylation in AAD[17]. Most recently, several studies had paid attention to the DNA methylation in AAD[18–20]. However, the DNA methylation patterns in AAD remain not completely clear. A two-stage study including the discovery stage and replication stage was designed to explore the DNA methylation profiles of AAD in this study.

## Methods

### Clinical samples

The study included a discovery stage and a replication stage. The discovery stage involved 4 AAD patients and 4 controls. The replication stage involved 16 AAD patients and 7 healthy controls independent of the patients in discovery stage. All the participants were recruited from 2011 to 2013 in Fuwai Hospital of the Chinese Academy of Medical Sciences in Beijing. Stanford-A AAD patients were diagnosed with typical symptoms and aortic computed tomography angiography. Individuals with Stanford-B AAD, Marfan syndrome, Loeys-Dietz Syndrome or familial aortic dissection were excluded. The healthy organ donors had no aortic diseases. The ascending aorta tissue samples of the participants were collected and frozen immediately in liquid nitrogen and then stored at -80℃. The study was approved by the Ethics Committee of Fuwai Hospital (Beijing, China) and complied with the Declaration of Helsinki. All the participants gave their informed consent.

### DNA isolation and bisulfite conversion

DNA was extracted from the aortic tissue samples using DNeasy Blood and Tissue Kit (Qiagen, Germany), then bisulfite-converted using EZ-DNA Methylation-Gold KIT (Zymo, USA) according to the manufacturer’s protocol.

### Infinium Human methylation 450 K assay

Infinium Human Methylation 450 K BeadChip (Illumina, USA) was used to detect DNA methylation. The β values represent the methylation levels of target sites which ranged between 0 (completely unmethylated) and 1 (completely methylated). The threshold of differentially methylated positions (DMPs) was defined as P-value ≤ 0.05 and |β difference (∆β)| ≥0.3. The analyses were conducted by Shanghai Biotechnology Corporation (Shanghai, China).

### Enrichment analyses

The enrichment analysis included Kyoto Encyclopedia of Genes and Genomes (KEGG) pathway and gene ontology (GO) analyses, which were performed using differentially methylated position-associated genes using KOBAS software[21].

### Bisulfite pyrosequencing

DNA was extracted, then bisulfite-converted and then amplified using polymerase chain reaction (PCR). Pyrosequencing reactions were performed using PyroMark Q24 (Qiagen, Germany). The primers were designed using PyroMark Assay Design Software 2.0 (Qiagen, Germany). The primers used for pyrosequencing were listed in Supplementary Table 1. All the experiments of pyrosequencing were conducted by Beijing Microread Genetic Corporation (Beijing, China) according to the manufacturer’s protocol.

### Western blot analysis

Aortic tissue samples were prepared in ice-cold cOmplete™ Lysis-M EDTA-free buffer (Roche, Germany) containing protease inhibitors. The lysed tissues were centrifuged and the supernatants were collected for Western blot analysis. The protein was separated by SDS polyacrylamide gels and transferred to polyvinylidene difluoride membranes. After blocked with 5% nonfat milk (BD, USA), the membranes were incubated with primary antibodies against Fas (Abcam,1:1000) or β-actin at 4℃ overnight. After washed with Tris buffered saline with Tween for 3 times, the membranes were incubated with the corresponding secondary antibodies at room temperature for 1 h. The membranes were washed for 3 times again and the protein bands were developed by chemiluminescence reagent (Millipore, USA).

### Data analyses

The statistical analyses were conducted with SPSS version 26.0 and GraphPad Prism 8 software. The Chi-square test was used to compare the categorical variables. Continuous variables were expressed as the mean ± standard. Student’s t-test and Mann-Whitney U test were used to compare the continuous variables of the two groups. P < 0.05 was considered statistically significant.

## Results

### Baseline characteristics of the participants

The flowchart showed the steps of our study (Supplementary Fig. 1). A total of 4 AAD patients and 4 healthy controls were recruited in the discovery cohort. The characteristics of participants were shown in Table 1. Participants in two groups were matched for gender and age. Only one AAD patient had the history of hypertension.

**Table 1:** Baseline characteristics of the participants for DNA methylation detection

	Infinium Human Methylation 450 K assay		Bisulfite pyrosequencing	
Variable	AAD (n = 4)	Controls (n = 4)	AAD (n = 16)	Controls (n = 7)
Age (years)	49.10 ± 4.91	47.92 ± 6.73	49.10 ± 4.52	47.81 ± 4.33
Male (n, %)	4(100)	4(100)	14(87.50)	5(71.43)
Hypertension (n, %)	1(25)	0(0)	13(81.25)	2(28.57)

Abbreviations: AAD, Acute aortic dissection

### DNA methylation analyses

**Fig. 1:**
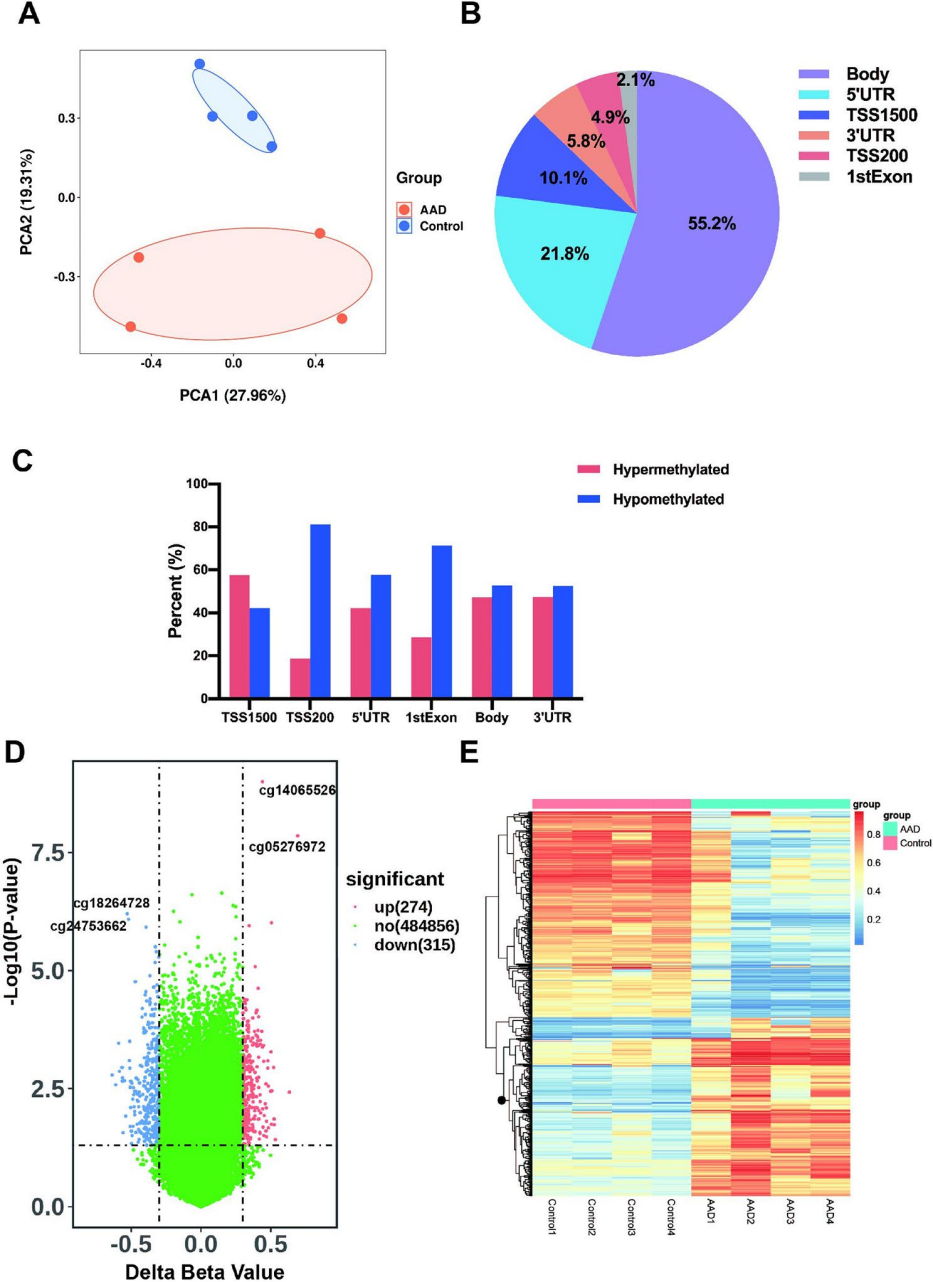
Visualization of the differential DNA methylation positions in aortic samples between Stanford-A acute aortic dissection and control group (**A**) Principal component analysis showing the differences of DNA methylation between the two groups (**B**) Percentages of differential DNA methylation in different genomic components (**C**) Hypermethylation and hypomethylation in different genomic components (**D**) Volcano plot presenting distribution and degree of the DNA methylation of the two groups. Red dots are the significantly hypermethylated positions and the blue dots are the significantly hypomethylated positions. The green dots are the positions with no significant difference. The top two significant hypermethylated and hypomethylated positions were labeled (**E**) Heat map showing different features of DNA methylation profile between the two groups

In the discovery stage, the overall differential DNA methylation patterns between Stanford-A AAD patients and healthy controls were assessed using Infinium Human Methylation 450 K BeadChip. Principal component analysis showed the differential DNA methylation patterns of the two groups (Fig. 1 A). According to the screening criteria (P-value ≤ 0.05 and |β difference (∆β)| ≥0.3), a total of 589 significantly differentially methylated positions were identified. About 55.2% of the DMPs were located in body region, 21.8% in 5’UTR and 10.1% in TSS1500 (Fig. 1B). We analyzed the distribution of all the methylated positions, and the results showed that most of the methylation positions were also located in the body regions (Supplementary Fig. 2). The distribution of hypermethylated and hypomethylated DMPs in different genomic components were shown in Fig. 1 C. More hypomethylated probes were found in TSS200, 5’UTR, 1stExon, body and 3’UTR than the hypermethylated probes. We also found DMPs in the intergenic region, including 126 hypermethylated probes and 137 hypomethylated probes. The volcano plot presented the distribution of 274 hypermethylated and 315 hypomethylated sites (Fig. 1D). The top two significantly hypermethylated positions including cg14065526 (P-value ≤ 0.001, ∆β = 0.44), cg05276972 (P-value ≤ 0.001, ∆β = 0.69) and the top two significantly hypomethylated positions including cg18264728 (P-value ≤ 0.001, ∆β=-0.53) and cg24753662 (P-value ≤ 0.001, ∆β=-0.52) were labeled in the volcano plot (Fig. 1D). The top 10 significantly hypermethylated, hypomethylated positions and the differentially methylated position-associated genes (DMP-associated genes) were listed in Tables 2 and 3, respectively. Of the genes associated with listed 20 most significant DMPs, NOTCH1 and LOXL2 were the genes associated with AAD. NOTCH1 was a hypermethylated position-associated gene related to cg14065526. The mutations in this gene were correlated with aortic aneurysm and dissection[22, 23]. Inhibition of NOTCH1 promoted β-aminopropionitrile-induced AD formation[24]. LOXL2 was a hypomethylated position-associated gene related to cg24531955 and was found upregulated in AAD[25]. The heat map was performed using the 589 DMPs, showing different features of DNA methylation profile between AAD patients and healthy controls, indicting the potential role of DNA methylation in distinguishing the two groups (Fig. 1E).

**Table 2:** Top 10 significant hypermethylated positions in Stanford-A AAD compared to healthy control

CpG site	Gene	Chr	Gene region	Location	△Beta	P values
cg14065526	NOTCH1	chr9	Body	S_Shore	0.440	<0.001
cg05276972					0.692	<0.001
cg26296371	FARS2		Body		0.505	<0.001
cg26707845	ODZ4		Body		0.345	<0.001
cg08224682					0.388	<0.001
cg03025413					0.410	<0.001
cg12524725					0.308	<0.001
cg06043710	DIP2C		Body		0.329	<0.001
cg14757661					0.316	<0.001
cg05877990					0.307	<0.001

Abbreviations: S-Shore, South shore which are regions flanking island; Chr, Chromosome. AAD, Acute aortic dissection

**Table 3:** Top 10 significant hypomethylated positions in Stanford-A AAD compared to healthy control

CpG site	Gene	Chr	Gene region	Location	△Beta	P values
cg18264728	DAB2	chr5	5’UTR	N_Shelf	-0.528	<0.001
cg24753662	TBX1	chr22	5’UTR	Island	-0.519	<0.001
cg03551963					-0.393	<0.001
cg24531955	LOXL2		3’UTR		-0.327	<0.001
cg04279411					-0.320	<0.001
cg18419977	SLC22A18AS	chr11	Body; TSS1500	N_Shelf	-0.302	<0.001
cg20597486	IFI16		5’UTR; 1stExon		-0.328	<0.001
cg15338782	ATP10A		Body		-0.365	<0.001
cg00903308		chr7		N_Shore	-0.470	<0.001
cg01134144	ARHGEF6		TSS200		-0.315	<0.001

Abbreviations: 5’UTR, 5’-Untranslated region; 3’UTR, 3’-Untranslated region; TSS1500, 1500 bp upstream of transcription start site; TSS200, 200 bp upstream of transcription start site; N-Shelf, North shelf which are regions flanking island; N-Shore, North shore which are regions flanking island; Chr, Chromosome. AAD, Acute aortic dissection

### Enrichment analyses

**Fig. 2:**
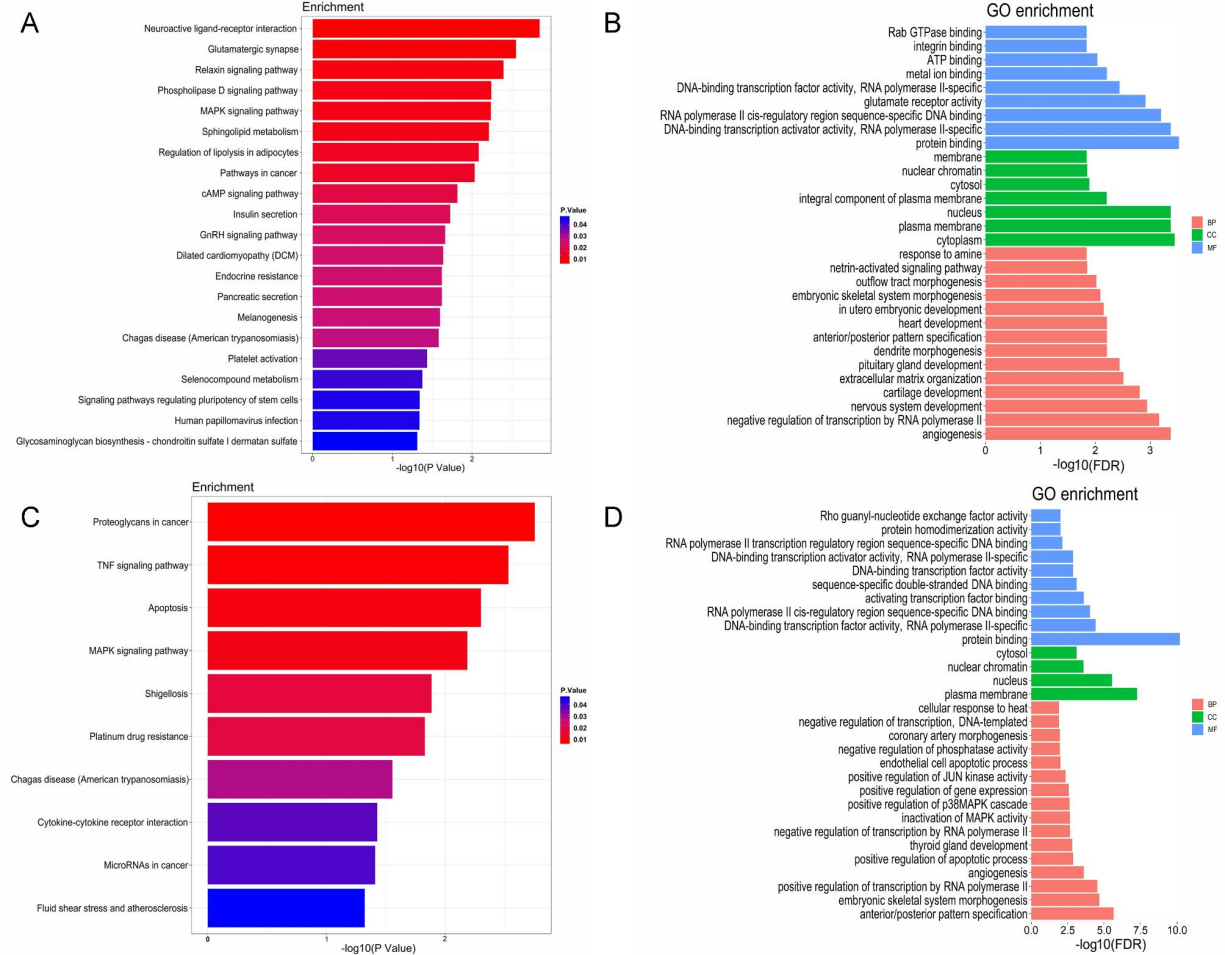
Bioinformatics analysis of hypermethylated position-associated genes (A, B) or hypomethylated position-associated genes (C, D) of the two groups (**A**) Significant Kyoto Encyclopedia of Genes and Genomes pathways of hypermethylated position-associated genes (P<0.05) (**B**) Top 30 significant enriched Gene Ontology terms of hypermethylated position-associated genes (P<0.05) (**C**) Significant Kyoto Encyclopedia of Genes and Genomes pathways of hypomethylated position-associated genes (P<0.05) (**D**) Top 30 significant enriched Gene Ontology terms of hypomethylated position-associated genes (P<0.05)

A total of 96 hypermethylated and 101 hypomethylated position-associated genes were found. Gene Ontology processes and Kyoto Encyclopedia of Genes and Genomes pathway enrichment were performed using DMP-associated genes. The hypermethylated position-associated genes were enriched in the KEGG pathways such as neuroactive ligand-receptor interaction, glutamatergic synapse, relaxin signaling pathway, phospholipase D signaling pathway, and MAPK signaling pathway, et al. (Fig. 2 A, Supplementary Table 2). The hypomethylated position-associated genes were enriched in proteoglycans in cancer, TNF signaling pathway, apoptosis, MAPK signaling pathway, and shigellosis, et al. (Fig. 2 C, Supplementary Table 3). The top 30 significantly enriched GO terms using hypermethylated (Fig. 2B, Supplementary Table 4) and hypomethylated (Fig. 2D, Supplementary Table 5) position-associated genes (P<0.05) were shown in Fig. 2. In GO analysis, hypermethylated position-associated genes were enriched in protein binding, angiogenesis and heart development et al. Hypomethylated position-associated genes were enriched in protein binding, angiogenesis and endothelial cell apoptotic process et al. The GO terms including angiogenesis, heart development and endothelial cell apoptotic process were the aortic dissection-associated terms, indicating the potential role of DNA methylation in the pathogenesis of aortic dissection. Hypermethylated and hypomethylated position-associated genes could be enriched in the same KEGG pathways or GO terms such as MAPK signaling pathway and protein binding, which indicated that they were associated with both hypermethylated and hypomethylated positions and played the key role in DNA methylation associated aortic dissection. MAPK signaling pathway was the significantly enriched pathway related to AAD and the DMP-associated genes including Fas, ANGPT2, and DUSP6 were enriched in it. Protein binding was the most significantly enriched GO term. DMP-associated genes including FARP1 and CARD6 were enriched in it.

### Differential DNA methylation validated by pyrosequencing

**Fig. 3:**
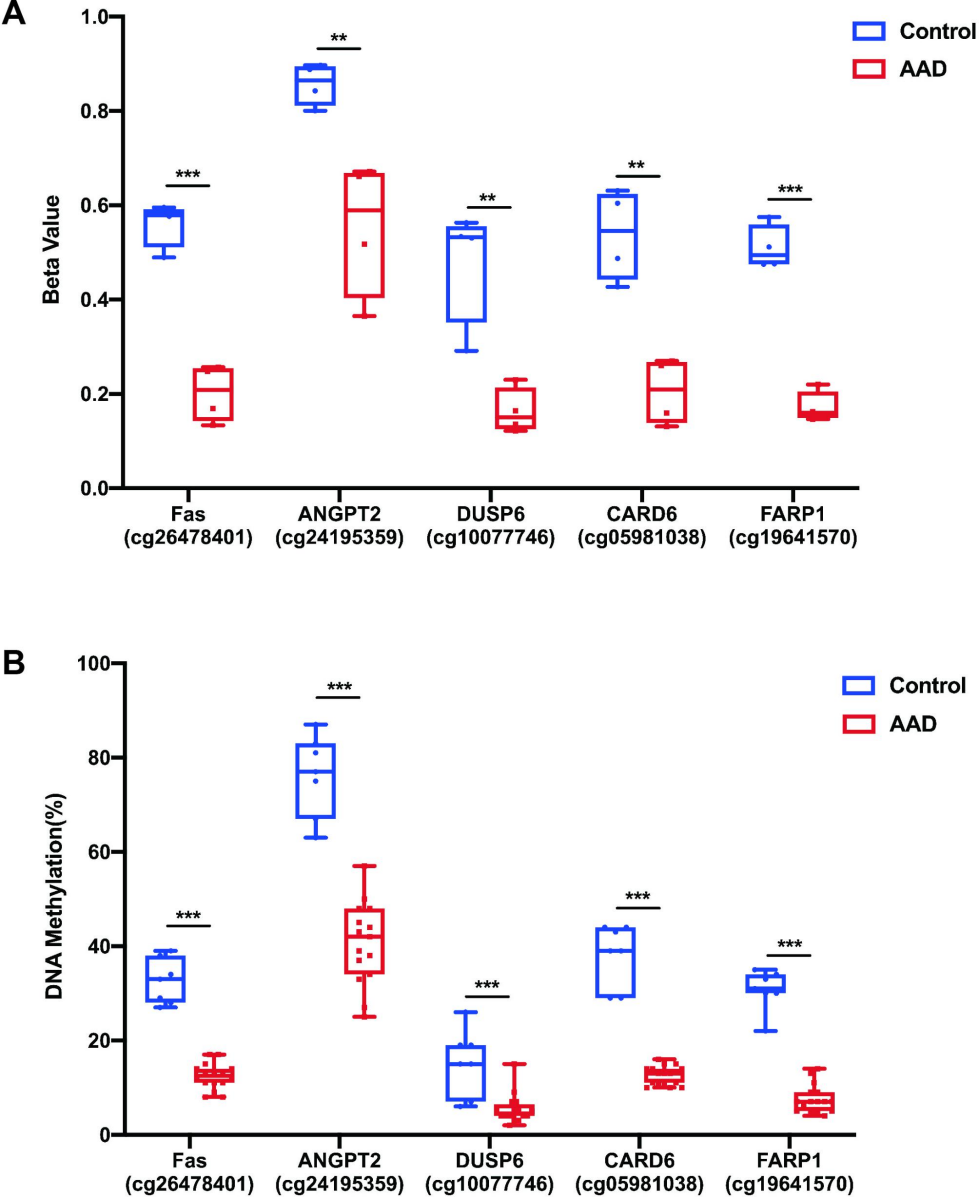
The results of Infinium Human methylation 450 K BeadChip (A) and pyrosequencing (B) of the five candidate genes including Fas, ANGPT2, DUSP6, FARP1, and CARD6

In the replication stage, we used pyrosequencing to validate the DNA methylation of AAD in an independent replication cohort including 16 AAD patients and 7 healthy controls. Based on our bioinformatics analyses, a total of 5 CpG dinucleotides covering 5 genes including ANGPT2, Fas, DUSP6, FARP1 and CARD6, were selected for pyrosequencing validation (Supplementary Table 6). The baseline characteristics of participants were shown in Table 1 and the participants were comparable in gender and age. The data in replication cohort were consistent with the results of discovery stage (Fig. 3), validating the differential DNA methylation profiles in AAD.

### The protein expression of Fas in AAD

**Fig. 4:**
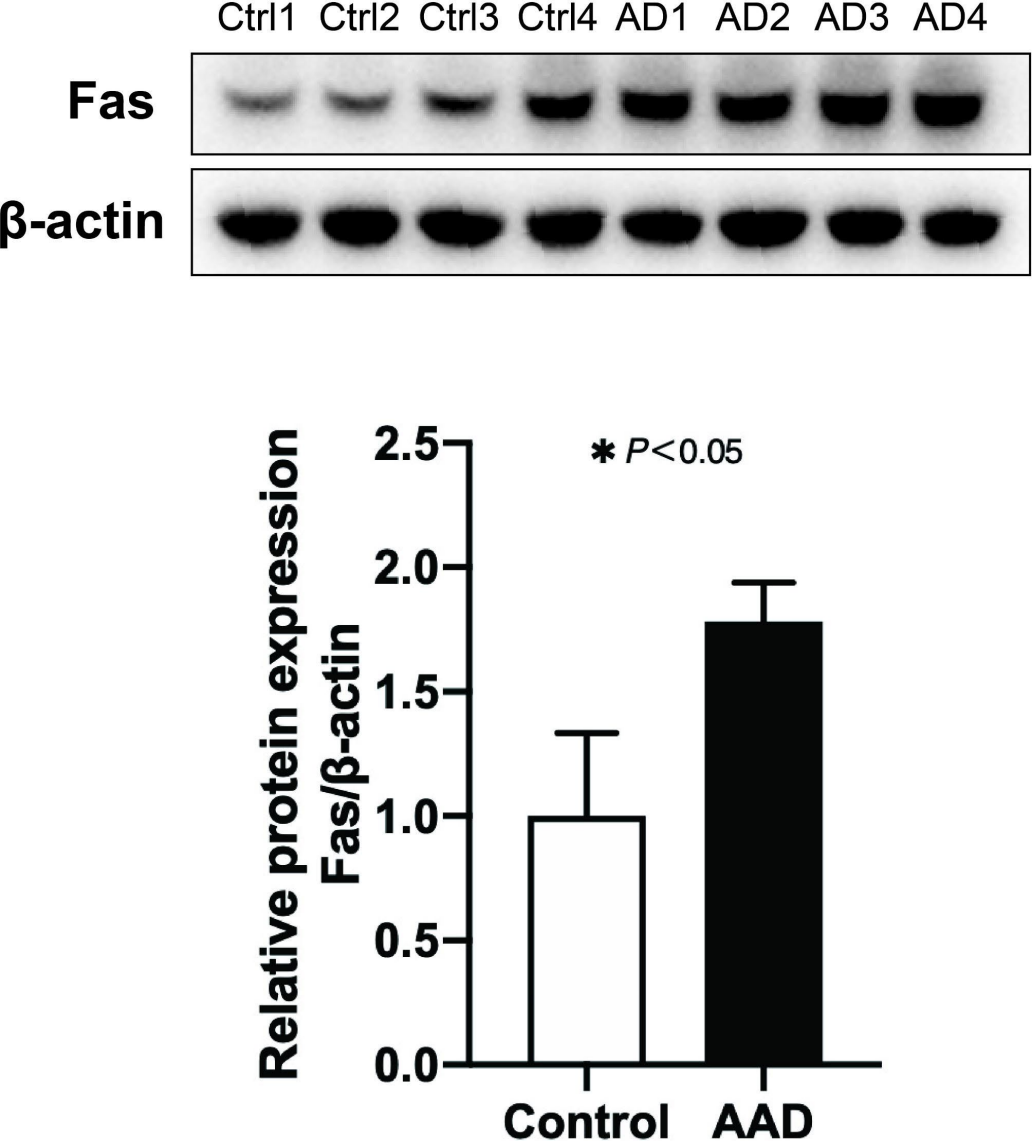
Validation of the protein expression level of Fas in aortic samples of acute aortic dissection (n = 4) and control group (n = 4) using Western Blot (Uncropped full-length western blot results are provided as the Supplementary Fig. 3). AAD, acute aortic dissection. AD, aortic dissection

In order to explore the potential association between DNA methylation and gene expression, we validated the protein expression of a selected DMP-associated gene in human aorta samples. The CpG site cg26478401 mapped to within 1500 bp of the TSS of Fas was significantly hypomethylated (P-value = 0.0001, ∆β= -0.36) in AAD group. In the previous studies, the protein expression of Fas was increased in AAD and AAA aorta samples[26, 27]. In line with these studies, we validated the increase of Fas in AAD in the present research (1.00 ± 0.34 vs. 1.78 ± 0.16, P<0.05, Fig. 4). The increased expression of the hypomethylated position-associated genes suggested the potential role of DNA methylation in regulating gene expression and the pathogenesis of AAD.

## Discussion

In this study, we used a two-stage exploration including discovery stage and replication stage to identify the DNA methylation profiles in Stanford-A AAD patients. A total of 589 differentially methylated positions including 315 hypomethylated and 274 hypermethylated positions were found in AAD group. KEGG analysis demonstrated that DMP-associated genes were enriched in MAPK signaling pathway, TNF signaling pathway and apoptosis pathway, et al. The differential DNA methylation of CpG sites in five key genes, including Fas, ANGPT2, DUSP6, FARP1 and CARD6, was authenticated in the independent replication cohort. The protein expression level of the hypomethylated position-associated gene Fas was increased by 1.78 times, indicating the possible role of DNA methylation in regulation of gene expression.

Previous studies found that DNA methylation played important roles in cardiovascular diseases, such as abdominal aortic aneurysm (AAA), atherosclerosis, coronary heart disease, hypertrophy, and arrhythmia[28–32]. In our previous research, it was found that the protein expression level of DNMT1 and DNMT3B decreased significantly in AAD patients while DNMT3A and DNMTL had the decreased trends with no significance, which indicated the potential role of DNA methylation in AAD[17]. Several previous studies also paid attention to the DNA methylation changes in AAD[18–20]. Li N et al. detected the DNA methylation in blood samples of TAD patients and found MMP2 promoter hypermethylated[18]. Liu P et al. compared the differential DNA methylation in ascending aortic tissues of thoracic aortic dissection (TAD) patients[19]. They found that the DMP-associated genes were enriched in vasculature and heart development, and Hox genes may be important in TAD pathogenesis[19]. The DNA methylation signature of plasma cell-free DNA in AAD was also detected[19]. Sun P et al. used 450k BeadChip for DNA methylation exploration and found that AAD was correlated with inflammatory vascular remodeling process, which was possibly related to environmental risk factors such as smoking[20].

In our study, MAPK signaling pathway was significantly enriched in KEGG pathway analysis using hypermethylated or hypomethylated position-associated genes. It is associated with cell proliferation, differentiation, metastasis, apoptosis, and the pathogenesis of aortic diseases[33–35]. Fas, ANGPT2 and DUSP6 were the AAD-related DMP-associated genes enriched in this pathway. In GO analysis, protein binding was the most significantly enriched GO terms and DMP-associated genes such as FARP1 and CARD6 were found in it. These five DMP-associated genes were selected for DNA methylation validation in the replication cohort in a larger number of samples. And the results were consistent with the data in discovery cohort. Fas plays a significant role in apoptosis[26, 27]. The protein level of Fas was found up-regulated in the aortic tissues of AAD and AAA patients in previous researches[26, 27]. And it was validated increased by 1.78 times in AAD group in our research. ANGPT2 is involved in the regulation of angiogenesis and inflammation and could be found in angiogenesis GO term enriched by hypomethylated position-associated genes[36, 37]. The elevated level of serum angiopoietin-2 was found in AAA men[37]. In addition, our previous study suggested the increased expression of ANGPT2 in AAD patients[38]. Another hypomethylated position-associated gene DUSP6 was also found up-regulated in AAA tissues[39]. Fas, ANGPT2 and DUSP6 were all the hypomethylated position-associated genes with increased gene expression in AAD. The results indicated a possible role of DNA methylation in regulating gene expression and promoting the progression of AAD, which needs to be validated in further study.

There are some limitations in this study. First of all, our sample sizes were relatively small (4 cases vs. 4 controls). The heterogeneity exists between samples within the same group may affect the experimental results. Also, to maximize the possibility to find more altered DNA methylation sites, weused unadjusted P value in our study. Thirdly, we did not integrate the differential DNA methylation with differentially expressed genes for analysis. In addition, we only validated the increased protein expression of Fas, while not pay attention to other selected candidate genes, which need to be further explored. Finally, the aorta tissues have multiple cell types such as vascular smooth muscle cells, endothelial cells and fibroblasts. It is important to identify the DNA methylation at the cell-specific level.

## Conclusion

In conclusion, this study explored the DNA methylation profiles in Stanford-A AAD, discovered the differentially methylated position-associated genes, enriched KEGG pathways including MAPK signaling pathway, TNF signaling pathway and apoptosis pathway, and GO terms including protein binding, angiogenesis and heart development et al. We also validated the increased protein expression of Fas. We proposed a hypothesis that DNA methylation may promote AAD by regulating expression of DMP-associated genes, which needs further exploration and validation.

## Electronic supplementary material

Below is the link to the electronic supplementary material.

Supplementary Material 1: DNA Methylation Alternation in Stanford-A Acute Aortic Dissection

## Data Availability

The datasets generated and/or analysed during the current study are available in the GEO repository, ​https://www.ncbi.nlm.nih.gov/geo/query/acc.cgi?acc=GSE202047​​​. To review GEO accession GSE202047, please enter token qrepsycmbdqnpsh into the box.

## Abbreviations

AAD: acute aortic dissection.
CpG: cytosine-paired-with-guanine.
DNMTs: DNA methyltransferases.
DMPs: differentially methylated positions.
KEGG: Kyoto Encyclopedia of Genes and Genomes.
GO: gene ontology.
DMP-associated genes: differentially methylated position-associated genes.
PCR: polymerase chain reaction.
TAD: thoracic aortic dissection.
AAA: abdominal aortic aneurysm.
