# Supplementary material for: DNA methylation alternation in Stanford- A acute aortic dissection
Source: BMC Cardiovasc Disord. 2022 Oct 29;22:455. doi: 10.1186/s12872-022-02882-5 (PMC9618190; doi:10.1186/s12872-022-02882-5)
Supplement: Supplementary file 1 — Supplementary Material 1: DNA Methylation Alternation in Stanford-A Acute Aortic Dissection [file 12872_2022_2882_MOESM1_ESM.docx]

**Supplementary Material**

**DNA Methylation Alternation in Stanford-A Acute Aortic Dissection**

Yufei Chen^1^, Xu Xu^1^, Zhaoran Chen^1,2^, Bi Huang^1,3^, Xiaojian Wang^4*^, Xiaohan Fan^1*^

^1^ Department of Cardiology, Fuwai Hospital, National Center for Cardiovascular Diseases, Chinese Academy of Medical Sciences, Peking Union Medical College, Beijing, China.

^2^ Department of Geriatrics and Gerontology, Beijing Friendship Hospital, Capital Medical University, Beijing, China

^3^ Department of Cardiology, The First Affiliated Hospital of Chongqing Medical University, Chongqing, China

^4^ Key Laboratory of Pulmonary Vascular Medicine, State Key Laboratory of Cardiovascular Disease, Fuwai Hospital, National Center for Cardiovascular

Diseases, Chinese Academy of Medical Sciences and Peking Union Medical College, Beijing, China

***Correspondence:** Xiaohan Fan, Department of Cardiology, Fuwai Hospital, National Center for Cardiovascular Diseases, Chinese Academy of Medical Sciences and Peking Union Medical College, No. 167, Beilishi Road, Xicheng District, Beijing 100037, China. Email address: fanxiaohan@fuwaihospital.org. Xiaojian Wang, Key Laboratory of Pulmonary Vascular Medicine, State Key Laboratory of Cardiovascular Disease, FuWai Hospital, National Center for Cardiovascular Diseases, Chinese Academy Medical Sciences and Peking Union Medical College, No. 167, Beilishi Road, Xicheng District, Beijing, 100037, China. E-mail address: wang_xiaojian@vip.163.com.

**Supplementary Figure 1**

**
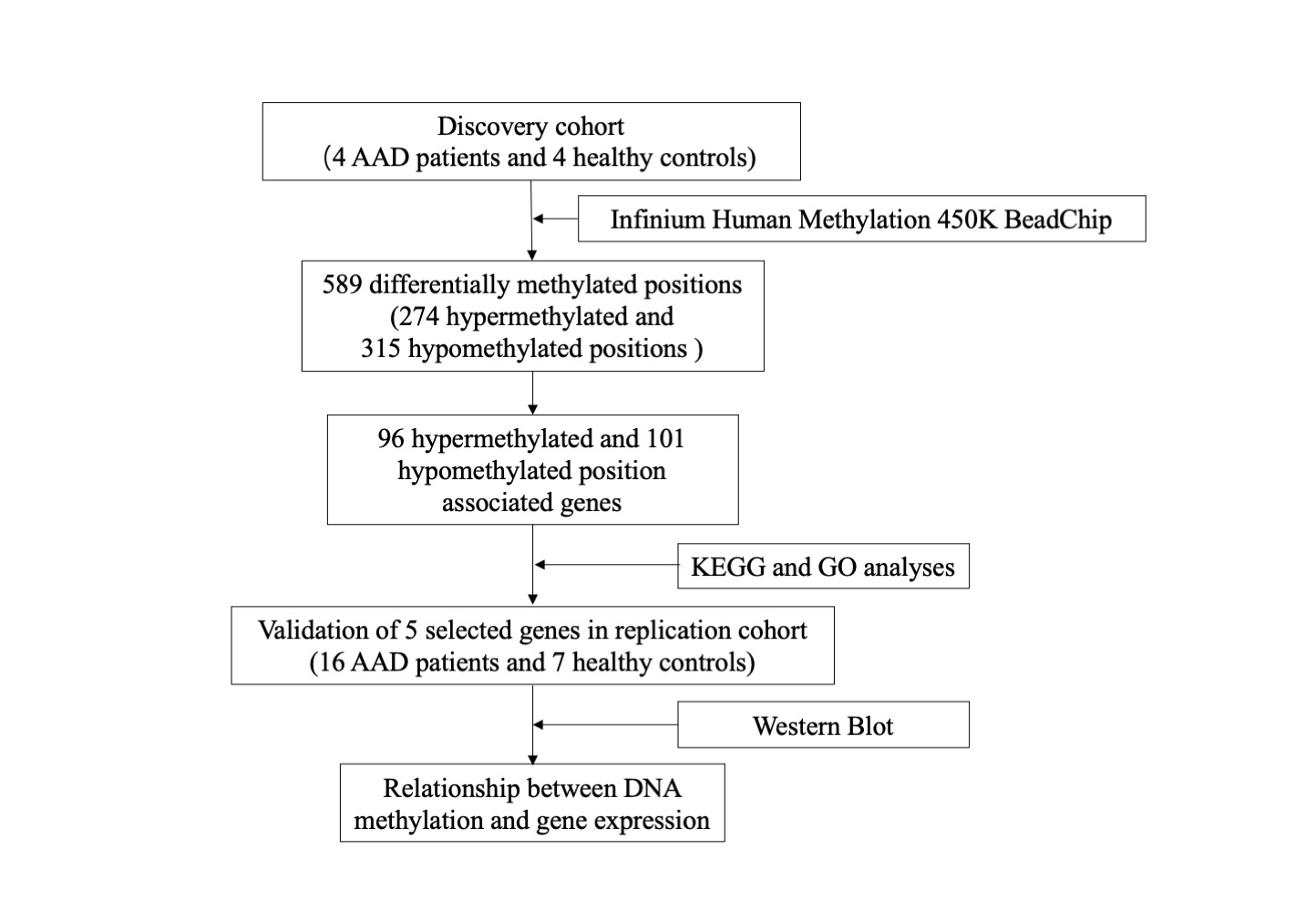
**

**Supplementary figure 1.** The flowchart of identificaion of differential methylation in this study. AAD, acute aortic dissection. DMPs, differentially methylated positions. KEGG, Kyoto Encyclopedia of Genes and Genomes. GO, gene ontology.

**Supplementary Figure 2**

**
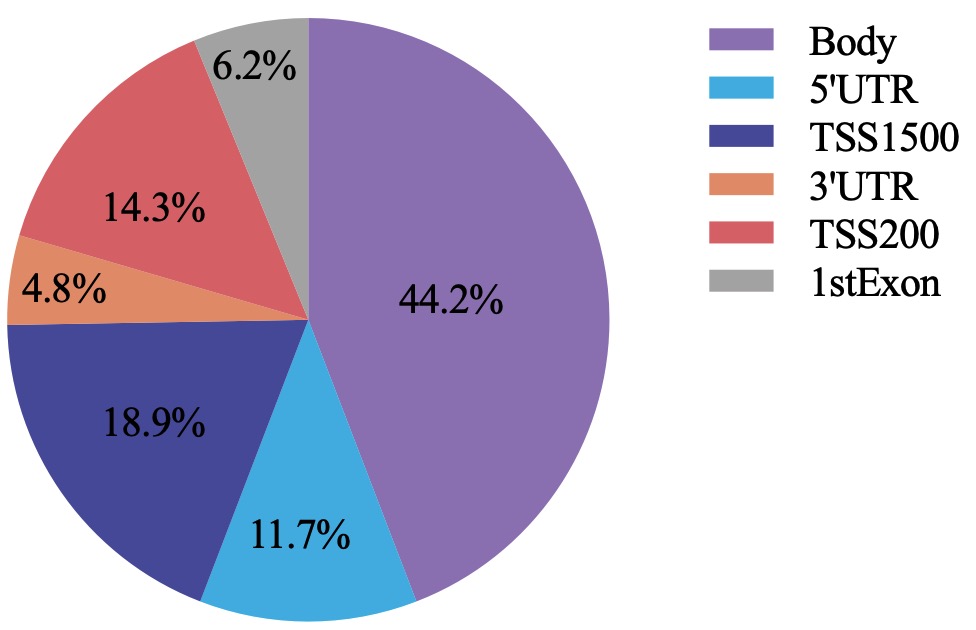
**

**Supplementary figure 2.** **Percentages of overall DNA methylation in different genomic components.**

**Supplementary Figure 3**

**
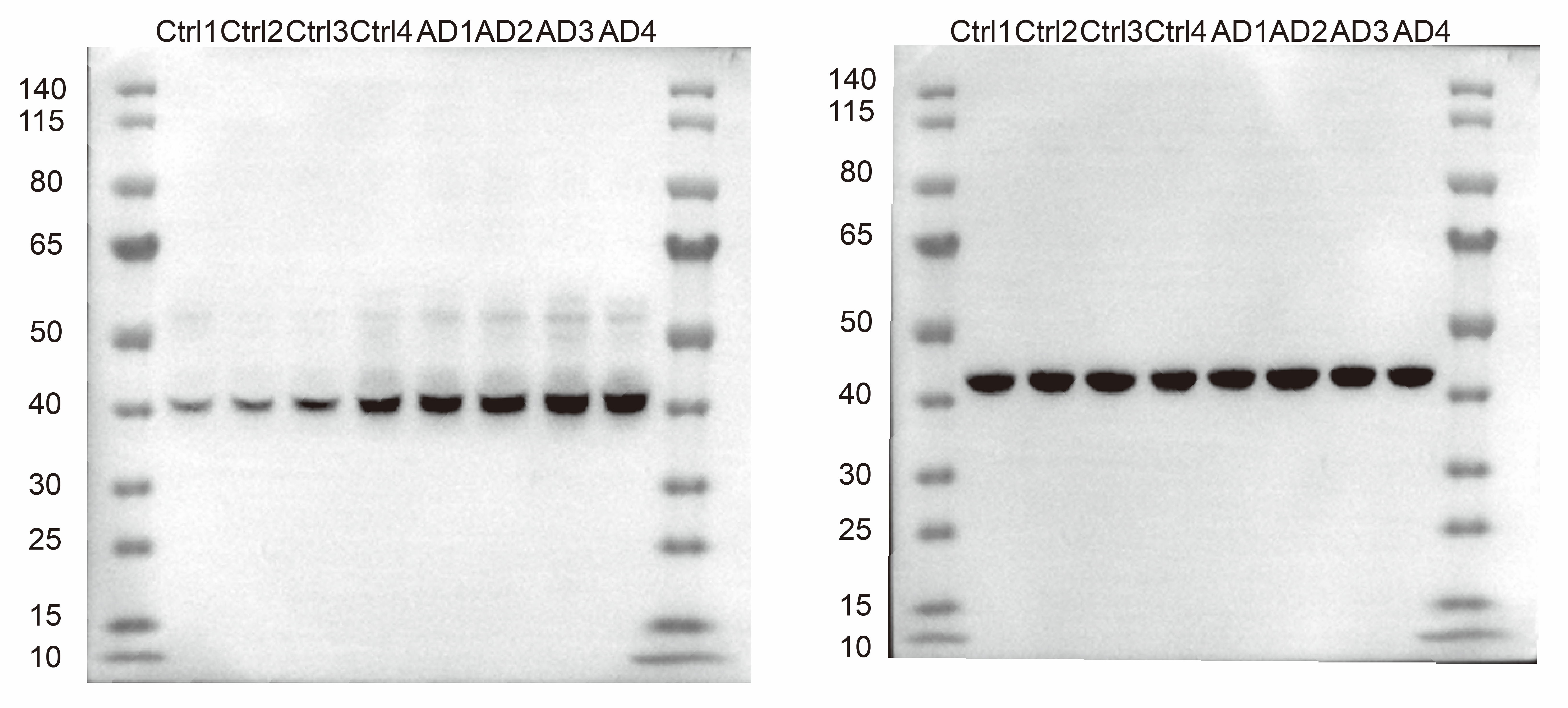
**

Fas β-actin

**Supplementary figure 3.** Uncropped full-length western blot results.

**Supplementary Table 1**

Primers used for pyrosequencing

| Gene | sequencing primer |
| --- | --- |
| ANGPT2 | GGTAGGGGAGGTAATGAGGAGCACCGATTAGGCTCCAAGATCCGT TCTGA |
| FAS | CCCTCCCTTTTCAGAGCCCTATGGCGCAACATCTGTACTTTTTCATATGG |
| FARP1  CARD6 | GGCCCTTGACATTCGTTTAAAGGAAGACARD6CGTTAACTTCTCGGAGGCTGATTTC  CGTTAACTTCTCGGAGGCTGATTTC |
| DUSP6 | ATTAGGAGGAACAGGATGTCGACACGGCCTGAAAATGAGTTCCTTTGTAA |

**Supplementary Table 2**

Significant enriched Kyoto Encyclopedia of Genes and Genomes pathways of hypermethylated position-associated genes (P＜0.05)

| Pathway ID | Pathway name | Gene list | P-Value |
| --- | --- | --- | --- |
| hsa04080 | Neuroactive ligand-receptor interaction | NPY1R, GRIK1, EDNRB, GRM8, GRID1 | 0.001 |
| hsa04724 | Glutamatergic synapse | GRIK1, GRM8, ADCY1 | 0.003 |
| hsa04926 | Relaxin signaling pathway | EDNRB, ADCY1, MAP2K4 | 0.004 |
| hsa04072 | Phospholipase D signaling pathway | GRM8, ADCY1, PPAP2B | 0.006 |
| hsa04010 | MAPK signaling pathway | MAPKAPK5, FLT1, NF1, MAP2K4 | 0.006 |
| hsa00600 | Sphingolipid metabolism | LASS3, PPAP2B | 0.006 |
| hsa04923 | Regulation of lipolysis in adipocytes | NPY1R, ADCY1 | 0.008 |
| hsa05200 | Pathways in cancer | GLI2, ITGA2, NOTCH1, EDNRB, ADCY1 | 0.009 |
| hsa04024 | cAMP signaling pathway | GPR81, NPY1R, ADCY1 | 0.015 |
| hsa04911 | Insulin secretion | ADCY1, RIMS2 | 0.019 |
| hsa04912 | GnRH signaling pathway | ADCY1, MAP2K4 | 0.022 |
| hsa05414 | Dilated cardiomyopathy (DCM) | ITGA2, ADCY1 | 0.023 |
| hsa04972 | Pancreatic secretion | RAB8A, ADCY1 | 0.024 |
| hsa01522 | Endocrine resistance | NOTCH1, ADCY1 | 0.024 |
| hsa04916 | Melanogenesis | EDNRB, ADCY1 | 0.025 |
| hsa05142 | Chagas disease (American trypanosomiasis) | ADCY1, MAP2K4 | 0.026 |
| hsa04611 | Platelet activation | ITGA2, ADCY1 | 0.037 |
| hsa00450 | Selenocompound metabolism | INMT | 0.042 |
| hsa04550 | Signaling pathways regulating pluripotency of stem cells | ISL1, HAND1 | 0.046 |
| hsa05165 | Human papillomavirus infection | ITGA2, NOTCH1, UBR4 | 0.046 |
| hsa00532 | Glycosaminoglycan biosynthesis - chondroitin sulfate / dermatan sulfate | CSGALNACT1 | 0.049 |

**Supplementary Table 3**

Significant enriched Kyoto Encyclopedia of Genes and Genomes pathways of hypomethylated position-associated genes (P＜0.05)

| Pathway ID | Pathway name | Gene list | P-Value |
| --- | --- | --- | --- |
| hsa05205 | Proteoglycans in cancer | COL21A1, CD44, MIR10B, FAS | 0.002 |
| hsa04668 | TNF signaling pathway | FAS, DAB2IP, MAP3K5 | 0.003 |
| hsa04210 | Apoptosis | FAS, DAB2IP, MAP3K5 | 0.005 |
| hsa04010 | MAPK signaling pathway | ANGPT2, FAS, DUSP6, MAP3K5 | 0.007 |
| hsa05131 | Shigellosis | CD44, DIAPH1 | 0.013 |
| hsa01524 | Platinum drug resistance | FAS, MAP3K5 | 0.015 |
| hsa05142 | Chagas disease (American trypanosomiasis) | FAS, TLR6 | 0.028 |
| hsa04060 | Cytokine-cytokine receptor interaction | FAS, ACVR1, IL17RA | 0.037 |
| hsa05206 | MicroRNAs in cancer | CD44, MIR10B, HDAC4 | 0.039 |
| hsa05418 | Fluid shear stress and atherosclerosis | ACVR1, MAP3K5 | 0.048 |

**Supplementary Table 4**

The top 30 significantly enriched Gene Ontology terms of hypermethylated position-associated genes

| ID | GO term | Gene list | P-Value |
| --- | --- | --- | --- |
| GO:0005515 | protein binding | AMOTL1, FARS2, LASS3, PPAP2B, COL11A2, CYFIP2, ISL1, ITGA2, RNF39, RBM9, ODZ4, HAND1, ATP11A, MAP2K4, XRCC1, DSCAM, EDNRB, CMIP, KIAA0182, DOCK11, MEOX2, FLT1, RAB8A, INMT, SIK3, C11orf49, BACH2, RSPH1, MAPKAPK5, A2BP1, GLI2, RPH3AL, NF1, FAM176A, RIMS2, HOXB2, C8orf46, PIK3C2B, EBF1, CORO2B, LCP1, GATA2, UBR4, ZFYVE21, NTM, NPY1R, NOTCH1, SCGN, PRDM6, EXD3, HOXA3, VAT1L | ＜0.001 |
| GO:0005737 | cytoplasm | FARS2, INMT, CYFIP2, ISL1, NAPEPLD, KIF26B, ODZ4, HAND1, RNF39, MAPKAPK5, A2BP1, MEOX2, SIK3, KIAA0802, NF1, RPH3AL, PIK3C2B, RBM9, UBR3, CORO2B, LCP1, GATA2, ADCY1, HRNBP3, ADARB2, SCGN, GRIP1, RNF213, HIVEP3 | ＜0.001 |
| GO:0001525 | angiogenesis | AMOTL1, HAND1, HOXB3, HOXA3, RNF213, MEOX2, FLT1 | ＜0.001 |
| GO:0001228 | DNA-binding transcription activator activity, RNA polymerase II-specific | HOXB2, HOXB3, NOTCH1, GLI2, HAND1, EBF1, ISL1, GATA2, MEOX2 | ＜0.001 |
| GO:0005886 | plasma membrane | AMOTL1, PPAP2B, KCNC2, ITGA2, NTM, ODZ4, ATP11A, DSCAM, EDNRB, FLT1, RAB8A, SLC6A18, GRM8, GPR81, SDK1, GRID1, C8orf46, OR6C68, PIK3C2B, UNC5D, LCP1, UBR4, ADCY1, GRIK1, NPY1R, NOTCH1, FAM176A, PRTG | ＜0.001 |
| GO:0005634 | nucleus | CYFIP2, ISL1, ITGA2, RBM9, ODZ4, HAND1, MAP2K4, XRCC1, MAPKAPK5, A2BP1, MEOX2, BACH2, RSPH1, GLI2, NF1, HOXB3, TAGLN3, HOXB2, C8orf46, EBF1, GATA2, ADCY1, HRNBP3, ADARB2, NOTCH1, SCGN, PRDM6, HOXA3, GRIP1, HIVEP3 | ＜0.001 |
| GO:0000978 | RNA polymerase II cis-regulatory region sequence-specific DNA binding | ISL1, HOXB2, HOXB3, NOTCH1, GLI2, HOXA3, HAND1, BACH2, GATA2, MEOX2 | ＜0.001 |
| GO:0000122 | negative regulation of transcription by RNA polymerase II | ISL1, HOXB2, HOXB3, NOTCH1, GLI2, PRDM6, HAND1, BACH2, EDNRB, TAGLN3, GATA2 | ＜0.001 |
| GO:0007399 | nervous system development | HRNBP3, GRIK1, DCLK1, RBM9, DSCAM, EDNRB, A2BP1 | ＜0.001 |
| GO:0008066 | glutamate receptor activity | GRIK1, GRM8, GRID1 | ＜0.001 |
| GO:0051216 | cartilage development | HOXB3, HOXA3, COL11A2, CSGALNACT1 | ＜0.001 |
| GO:0030198 | extracellular matrix organization | ADAMTS17, COL11A2, THSD4, NF1, ITGA2, CSGALNACT1 | ＜0.001 |
| GO:0021983 | pituitary gland development | GLI2, ISL1, GATA2 | ＜0.001 |
| GO:0000981 | DNA-binding transcription factor activity, RNA polymerase II-specific | ISL1, HOXB2, HOXB3, GLI2, HOXA3, HAND1, EBF1, BACH2, GATA2, MEOX2 | ＜0.001 |
| GO:0048813 | dendrite morphogenesis | DSCAM, DCLK1, RBM9 | ＜0.001 |
| GO:0009952 | anterior/posterior pattern specification | GLI2, HOXB3, HOXA3, HOXB2 | ＜0.001 |
| GO:0007507 | heart development | GLI2, ISL1, NOTCH1, NF1, HAND1 | ＜0.001 |
| GO:0046872 | metal ion binding | ZFYVE21, ADCY1, COL11A2, RPH3AL, ISL1, ITGA2, ADARB2, PRDM6, EXD3, RIMS2, ADAMTS17, RNF39, EBF1, CSGALNACT1, RNF213, HIVEP3 | ＜0.001 |
| GO:0005887 | integral component of plasma membrane | ADCY1, KCNC2, SLC6A18, GRIK1, NPY1R, DCLK1, ODZ4, GRM8, DSCAM, EDNRB, PPAP2B, FLT1 | ＜0.001 |
| GO:0001701 | in utero embryonic development | GLI2, UBR3, CMIP, NOTCH1, ATP11A | ＜0.001 |
| GO:0048704 | embryonic skeletal system morphogenesis | HOXB2, HOXB3, HOXA3 | ＜0.001 |
| GO:0005524 | ATP binding | DNAH2, FARS2, ADCY1, SIK3, DCLK1, TTLL8, KIF26B, PIK3C2B, ATP11A, MAP2K4, MAPKAPK5, FLT1 | ＜0.001 |
| GO:0003151 | outflow tract morphogenesis | ISL1, NPY1R, NOTCH1 | ＜0.001 |
| GO:0005829 | cytosol | AMOTL1, INMT, CYFIP2, CLEC16A, NAPEPLD, RBM9, MAP2K4, MAPKAPK5, CMIP, DOCK11, RAB8A, BACH2, RSPH1, GLI2, NF1, NGEF, HOXB2, TTLL8, PIK3C2B, LCP1, UBR4, OSBPL10, NOTCH1, SCGN, RNF213 | ＜0.001 |
| GO:0038007 | netrin-activated signaling pathway | DSCAM, UNC5D | ＜0.001 |
| GO:0000790 | nuclear chromatin | ISL1, HOXB2, HOXB3, HOXA3, HAND1, EBF1, BACH2, XRCC1, MEOX2 | ＜0.001 |
| GO:0005178 | integrin binding | GRIP1, ITGA2, LCP1, PPAP2B | ＜0.001 |
| GO:0014075 | response to amine | ITGA2, KCNC2 | ＜0.001 |
| GO:0016020 | membrane | OSBPL10, PPAP2B, NGEF, ATP11A, KCNC2, CYFIP2, GLI2, PIK3C2B, NF1, CORO2B, DSCAM, GCNT3, RNF213, UBR4 | ＜0.001 |
| GO:0017137 | Rab GTPase binding | RPH3AL, CLEC16A, RAB8A, RIMS2 | ＜0.001 |

**Supplementary Table 5**

The top 30 significantly enriched Gene Ontology terms of hypomethylated position-associated genes

| ID | GO term | Gene list | P-Value |
| --- | --- | --- | --- |
| GO:0005515 | protein binding | BAG3, ZNF217, ACVR1, PIWIL2, ATP11A, SKAP2, HOXC4, SPRED2, PRR5, POLE, TBX1, MAB21L1, WDR81, HAND2, DUSP14, DAB2, NEK9, MYOF, HDAC4, LOXL2, SORBS2, KRT80, FAS, FARP1, ARHGEF3, MAP3K5, ADAP1, ARHGAP22, IFI16, RARRES1, CD44, ANGPT2, DUSP6, CARD6, TMEM51, FAM98B, DIAPH1, TFEC, ARHGEF6, HOXD4, ATP10A, EMP1, DAB2IP, HOXD3, NR2F2, CYTH1, KIRREL3, GATA5, CSGALNACT2, NPFFR1, SRPK2, RGS17, ANKLE2, ITK, WDR90, RELL1, FAM124B, THADA, PDE4DIP, HOXA5, TLR6, GPR98, ASCC2, EFR3B, IL17RA | ＜0.001 |
| GO:0005886 | plasma membrane | BAG3, ACVR1, SLC6A6, ATP11A, SKAP2, SPRED2, POLE, CDSN, ABHD3, DAB2, SORBS2, MYOF, FAS, ADAP1, CD44, ANGPT2, CDH4, EFR3B, SDK1, SLITRK2, DIAPH1, RIMBP2, ATP10A, EMP1, DAB2IP, MUC4, CYTH1, KIRREL3, NPFFR1, RGS17, GPR158, RELL1, TLR6, GPR98, BEST3, IL17RA | ＜0.001 |
| GO:0009952 | anterior/posterior pattern specification | HOXD4, HOXC4, NR2F2, HOXA6, HOXA5, TBX1, HOXD3 | ＜0.001 |
| GO:0005634 | nucleus | BAG3, ZNF217, PIWIL2, HOXC4, POLE, TBX1, HAND2, SORBS2, HDAC4, LOXL2, PDE4DIP, MAB21L1, NEK9, ADAP1, ARHGAP22, IFI16, ANGPT2, SRPK2, POU3F3, FAM98B, DIAPH1, HOXD4, HOXD3, NR2F2, GATA5, NKX6-1, RGS17, RFX8, ITK, HRNBP3, PPAN, ADARB2, HOXA6, HOXA5, ASCC2 | ＜0.001 |
| GO:0048704 | embryonic skeletal system morphogenesis | HOXD4, HOXA6, HOXA5, HOXC4, HOXD3 | ＜0.001 |
| GO:0045944 | positive regulation of transcription by RNA polymerase II | POU3F3, ACVR1, HDAC4, TFEC, HOXD4, HOXC4, DAB2IP, IFI16, HOXD3, HOXA5, TBX1, HAND2, DAB2, GATA5, NKX6-1 | ＜0.001 |
| GO:0000981 | DNA-binding transcription factor activity, RNA polymerase II-specific | POU3F3, RFX8, TFEC, HOXD4, HOXC4, HOXD3, NR2F2, HOXA6, HOXA5, TBX1, HAND2, GATA5, NKX6-1 | ＜0.001 |
| GO:0000978 | RNA polymerase II cis-regulatory region sequence-specific DNA binding | HOXC4, ZNF217, RFX8, HDAC4, TFEC, IFI16, HOXD3, HOXA6, HOXA5, GATA5, NKX6-1 | ＜0.001 |
| GO:0001525 | angiogenesis | ACVR1, DAB2IP, ARHGAP22, TBX1, HAND2, ANGPT2, SRPK2 | ＜0.001 |
| GO:0033613 | activating transcription factor binding | HOXD4, HDAC4, HAND2, HOXC4 | ＜0.001 |
| GO:0000790 | nuclear chromatin | POU3F3, RFX8, TFEC, HOXD4, HOXC4, HOXD3, HOXA6, HOXA5, TBX1, HAND2, GATA5, NKX6-1 | ＜0.001 |
| GO:0005829 | cytosol | BAG3, HDAC4, SKAP2, SPRED2, PRR5, WDR81, DAB2, NEK9, ARHGEF6, FAS, ARHGEF3, NR2F2, ADAP1, ARHGAP22, IFI16, DUSP6, THADA, EFR3B, DIAPH1, FARP1, CD44, DAB2IP, CYTH1, MAN1A1, SRPK2, ITK, MAP3K5, KRT80, COL21A1 | ＜0.001 |
| GO:1990837 | sequence-specific double-stranded DNA binding | TFEC, HOXD4, HOXC4, HOXD3, HOXA6, HOXA5, TBX1, HAND2, GATA5 | ＜0.001 |
| GO:0003700 | DNA-binding transcription factor activity | POU3F3, ZNF217, RFX8, HOXD3, NR2F2, HOXA5, TBX1, GATA5, NKX6-1 | ＜0.001 |
| GO:0043065 | positive regulation of apoptotic process | ARHGEF6, FAS, DAB2IP, ARHGEF3, MAP3K5, HOXA5, DUSP6 | ＜0.001 |
| GO:0001228 | DNA-binding transcription activator activity, RNA polymerase II-specific | POU3F3, TFEC, HOXD4, HOXC4, HOXD3, HOXA5, TBX1, HAND2 | ＜0.001 |
| GO:0030878 | thyroid gland development | HOXA5, TBX1, HOXD3 | ＜0.001 |
| GO:0000122 | negative regulation of transcription by RNA polymerase II | ZNF217, HDAC4, TFEC, DAB2IP, LOXL2, NR2F2, IFI16, TBX1, DAB2, NKX6-1 | ＜0.001 |
| GO:0000188 | inactivation of MAPK activity | DUSP14, DUSP6, SPRED2 | ＜0.001 |
| GO:1900745 | positive regulation of p38MAPK cascade | RELL1, HAND2, MAP3K5 | ＜0.001 |
| GO:0010628 | positive regulation of gene expression | POU3F3, FAM98B, HOXD3, TLR6, HAND2, GATA5, SRPK2 | ＜0.001 |
| GO:0043507 | positive regulation of JUN kinase activity | TLR6, DAB2IP, MAP3K5 | ＜0.001 |
| GO:0000977 | RNA polymerase II transcription regulatory region sequence-specific DNA binding | HOXD4, HOXC4, NR2F2, TBX1, HAND2, HOXD3 | ＜0.001 |
| GO:0042803 | protein homodimerization activity | POU3F3, ACVR1, CDSN, DAB2IP, MAP3K5, TBX1, HAND2, NR2F2 | ＜0.001 |
| GO:0072577 | endothelial cell apoptotic process | DAB2IP, MAP3K5 | ＜0.001 |
| GO:0005089 | Rho guanyl-nucleotide exchange factor activity | ARHGEF6, ARHGEF3, FARP1 | ＜0.001 |
| GO:0010923 | negative regulation of phosphatase activity | WDR81, RIMBP2, FARP1 | ＜0.001 |
| GO:0060982 | coronary artery morphogenesis | TBX1, HAND2 | ＜0.001 |
| GO:0045892 | negative regulation of transcription, DNA-templated | POU3F3, LOXL2, HDAC4, DAB2IP, ZNF217, NR2F2, IFI16 | ＜0.001 |
| GO:0034605 | cellular response to heat | BAG3, MYOF, TFEC | ＜0.001 |

**Supplementary Table 6**

The 450k BeadChip results of the five candidate genes selected for pyrosequencing.

| \| CpG site \| Gene \| Chr \| Gene region \| Location \| △Beta \| P values \| \| --- \| --- \| --- \| --- \| --- \| --- \| --- \| \| cg24195359 \| ANGPT2 \| 8 \| Body \| - \| -0.3032 \| 0.0070 \| \| cg26478401 \| FAS \| 10 \| TSS1500 \| N-Shore \| -0.3588 \| 0.0001 \| \| cg19641570 \| FARP1 \| 13 \| 5’UTR \| N-Shelf \| -0.3380 \| 0.0000 \| \| cg05981038 \| CARD6 \| 5 \| 1stExon \| - \| -0.3324 \| 0.0014 \| \| cg10077746 \| DUSP6 \| 12 \| Body \| N-Shore \| -0.3166 \| 0.0034 \| |
| --- | --- | --- | --- | --- | --- | --- | --- | --- | --- | --- | --- | --- | --- | --- | --- | --- | --- | --- | --- | --- | --- | --- | --- | --- | --- | --- | --- | --- | --- | --- | --- | --- | --- | --- | --- | --- | --- | --- | --- | --- | --- | --- |

Abbreviations: TSS1500, 1500bp upstream of transcription start site; 5’UTR, 5’-Untranslated region; N-Shore, North shore which are regions flanking island; N-Shelf, North shelf which are regions flanking island; Chr, Chromosome. AAD, acute aortic dissection.
